# Hotel Guests’ Psychological Distance of Climate Change and Environment-Friendly Behavior Intention

**DOI:** 10.3390/ijerph19010016

**Published:** 2021-12-21

**Authors:** Wansoo Kim, Chen Che, Chul Jeong

**Affiliations:** 1 Department of Tourism Management, Gachon University, 1342 Seongnam-daero, Sujeong-gu, Incheon 13120, Korea; warooo@gachon.ac.kr; 2 College of History and Tourism Culture, Inner Mongolia University, No. 235, Daxue West Road, Hohhot 010021, China; chechen@imu.edu.cn; 3 Division of Tourism Science, Hanyang University, 222 Wangsimni-ro, Seongdong-gu, Seoul 04763, Korea

**Keywords:** global identity, psychological distance of climate change, norm activation model, hotel, environment-friendly behavior

## Abstract

Climate change is certainly a global problem that negatively affects all nations, and thus all humans, on the globe. Nevertheless, little is known about people’s perceptions of climate change and its effects on people’s attitudinal and behavioral responses to climate change. The present study successfully addressed how hotel guests’ environment-friendly behavior intention is formed through their self-perception as a member of the global community and their psychological distance of climate change. An online survey was used to collect quantitative data from hotel guests to verify the hypotheses. Our test results supported all the hypotheses in our conceptual model. Consequently, the findings of this study satisfactorily explained how hotel guests form their intention to engage in environment-friendly behaviors while they are staying at hotels.

## 1. Introduction

To make tourism on the globe sustainable in the future, it is critical for every participant to be faithful to one’s role. In the lodging industry, the environment-friendly management of hotels, together with the environment-friendly behaviors of guests, is commonly regarded as the core element of green management [1]. Making hotel operations more environment-friendly is also considered one of the optimal marketing strategies, especially in the developed countries, given the growing green consciousness in the mature lodging markets [2]. In addition to that, to be truly environment-friendly hotels, the active engagement of hotel guests in pro-environmental behaviors (e.g., water saving, waste reduction, and towel/linen reuse) is necessary [1,3].

However, psychological distance has been often identified as a major barrier to act on climate change [4]. The psychological distance of climate change leads individuals to feel as if climate change is not a pressing issue, irrelevant to where they live and to whom they know personally, and an unlikely event [5]. Hotel managements have clear financial reasons to manage hotels in an environment-friendly manner [6] because, as mentioned above, it is a useful marketing strategy and thus more profitable. On the other hand, hotel guests are different. They are typically not directly rewarded by behaving environment-friendly while staying at hotels. That means, for most guests, behaving environment-friendly is going beyond their self-interests. In this sense, environment-friendly behaviors have been treated as altruistic or prosocial behaviors in the literature [7]. However, when hotel guests perceive that climate change is currently and directly harming them and their family (i.e., those psychologically close to them), it is a different story. Then, behaving environment-friendly is no longer beyond their self-interests, but within their self-interests. It is because feeling psychologically close to climate change means accepting climate change as an urgent issue to oneself [8]. The previous studies typically only researched environment-friendly behavior intention and the customer’s prosocial behaviors [1,6,7]. However, there is no previous research in the literature on the psychological distance of climate change. Hence, this research aims to extend the norm activation model (NAM) to explain the psychological distance of climate change and environment-friendly behavior intention.

Accordingly, this study adopted the psychological distance of climate change as a key antecedent of the environment-friendly behavior intention of hotel guests. In doing so, this study used the norm activation model [9] as a conceptual framework. The NAM has been extensively applied in the explanation of various prosocial behaviors, including environment-friendly behaviors [7,10]. However, different from previous studies, this study did not use the NAM in its original form, but replaced awareness of consequences (AC) with psychological distance of climate change. Chen [11] investigated whether the psychological distance of climate change directly influenced people’s environmental concerns. Furthermore, simply being aware of the undesirable outcomes of not conducting certain behaviors, which is AC in the NAM, is not precise enough to predict people’s behavior intention if they are not sure when, where, to whom, and with how much probability the negative consequences are to be expected. Meanwhile, the psychological distance of climate change directly deals with those critical aspects of the negative consequences of climate change. Furthermore, this study adopted global identity as a significant predictor of the psychological distance of climate change, since climate change is a global issue certainly affecting all humans on the globe.

## 2. Literature Review and Hypotheses

### 2.1. Global Identity and Psychological Distance of Climate Change

Based on social identity theory (SIT), global identity can be defined as identifying oneself with all humans [12], that is, recognizing oneself as a member of the human race. The definition of global identity can be extended to include people’s emotional bonds, not only to human beings but also to the world or globe [13,14]. It is a general belief that humans are from mother nature. It means that people are inherently connected not only to human society but also to the earth or world. Therefore, it is natural to define global identity as identifying oneself with the world as well as its people. This definition is supported by the notion of location-based identification [15], with which people develop a feeling of identity from their sense of belonging to geographical groups, such as a country [16]. In sum, global identity represents a sense of belonging to the world and human society.

Construal level theory explains that individuals variously perceive events and objects based on their self-relevance and likelihood [17]. According to the theory, if considered psychologically distant, things are perceived as relatively abstract, whereas if considered close, things are perceived as relatively concrete. Four dimensions of psychological distance have been defined, namely temporal distance (i.e., whether an event is near or far in time), spatial distance (i.e., whether an event is geographically close or far away), social distance (i.e., whether an event will happen to oneself, socially close others, or socially distant others), and hypothetical distance (i.e., whether an event is likely/unlikely to happen) [18]. Therefore, the psychological distance of climate change represents individuals’ perceptions of the temporal, spatial, social, and hypothetical distances of climate change. For instance, if people think that the harm caused by climate change will occur tomorrow (i.e., temporally close distance), in my town (i.e., spatially close distance), to my family (i.e., socially close distance), and with 90% certainty (i.e., hypothetically close distance), they will perceive the harm as psychologically close to them [17], leading them to take actions to minimize the negative impacts of climate change. Unfortunately, however, a large portion of people on the earth seem to perceive climate change as psychologically distant, resulting in the lack of action on climate change [5].

Again, global identity refers to recognizing oneself as a part of not only all humans but also the world. The literature shows that those who have a strong global identity tend to actively engage in actions that are favorable to the earth and its people [14]. For human beings, it is natural to protect their community since they are a part of it. It is because protecting their community should be felt as being directly related to their survival in it [19]. Thus, those who have a strong global identity would be more concerned with the dangers of climate change on earth. Thus, for them, it would be not so difficult to realize that more and more frequent severe weather phenomena due to climate change are taking place currently (i.e., temporally close), all over the world (i.e., spatially close), thus indiscriminately to anyone (i.e., socially close), and with certainty (hypothetically close) [5]. In the last few years, there has been an increasing amount of literature on the psychological distance of climate change [11,20]. Loy and Spence [20] identify the relationship between people’s global identity and the psychological distance of climate change. Therefore, those who have stronger global identity would perceive less psychological distance of climate change.

**Hypothesis** **1** **(H1).** *Global identity negatively affects psychological distance of climate change.*

### 2.2. Ascribed Responsibility, Personal Norm, and Prosocial Behavior

The norm activation model (NAM) [9] describes a mechanism through which people form intentions for prosocial behaviors, including environment-friendly behaviors [7,10]. This theoretical model includes ascribed responsibility as a predictor of personal norm that affects prosocial behavior intention [9]. Ascribed responsibility represents the perception of shared responsibility for the harmful outcomes caused by not performing prosocial behaviors [21]. Radic et al. [10] outlined that the customer’s pro-environment behavior was related to an ascribed responsibility to anticipate guilt and anticipate pride. Kiatkawsin et al. [22] concluded that savvy tourists’ ascribed responsibility positively influenced environmental behavior. As such, ascribed responsibility is for something bad or wrong. Thus, if people perceive the negative consequences of climate change as psychologically distant, namely, that they perceive that the events will happen in the remote future, in other countries, to someone who they do not know, and with a low probability, the negative consequences would not be salient to them and thus trigger only minimal ascribed responsibility, if any. Therefore, the following hypothesis can be set.

**Hypothesis** **2** **(H2).** *Psychological distance of climate change negatively affects ascribed responsibility for not acting environment-friendly.*

Personal norm, as the central element in the NAM, indicates a moral obligation for performing prosocial behaviors [23]. According to the NAM, a feeling of moral obligation leads to altruistic behavior. This perception of obligation can be offset by defensive cognition about the appropriateness or relevance of the obligation to an event [9]. To determine the effects of the personal norm, Kiatkawsin et al. [22] tested attitude as a mediation factor to an indirect result of the personal norm and environmental responsibility behavior intention. Thus, taking the NAM and the construal level theory [17] together, it is plausible to say that personal norm cannot be activated if individuals cannot vividly construe the phenomena of climate change due to their far psychological distance of climate change. It is because if the phenomena are not vivid enough in people’s perception, they would doubt the appropriateness or relevance of their obligation to the phenomena, and thus feel less obligation to perform environment-friendly behaviors. Therefore, the following hypothesis would be valid.

**Hypothesis** **3** **(H3).** *Psychological distance of climate change negatively affects personal norm for environment-friendly behavior.*

Through the norm activation theory, Schwartz [9] explains that it is only when individuals accept their responsibility for the outcomes of not acting in a certain way, that they tend to form a personal norm (i.e., moral obligation) to act in a certain way. That is, people first feel a *sense* of responsibility, and then this sense induces people to develop an *obligation* toward a certain behavior depending on their judgement about whether it is the right thing to do (or not) from their value-based conceptions, which define how people should or should not behave in certain situations [24]. Then, the *obligation* leads people to engage in certain *behaviors* [9]. Engaging in certain behaviors can cause some costs or sacrifices to people, especially when the behaviors are not directly beneficial to them. Personal norms have been affirmed as major contributing factors for the green behavior of tourists [25]. Han et al. [26] confirmed that for young travelers, as a growing portion of international tourists, personal norm primarily influences their environmental behaviors (e.g., waste reduction, and recycling). Thus, individuals should perceive an obligation as a condition to behave prosocially, regardless of their self-interests [9]. Therefore, the following hypotheses can be established.

**Hypothesis** **4** **(H4).** *Ascribed responsibility for not acting environment-friendly positively affects personal norm for environment-friendly behavior.*

**Hypothesis** **5** **(H5).** *Personal norm for environment-friendly behavior positively affects environment-friendly behavior intention (waste reduction intention, water saving intention).*

**Figure 1:**
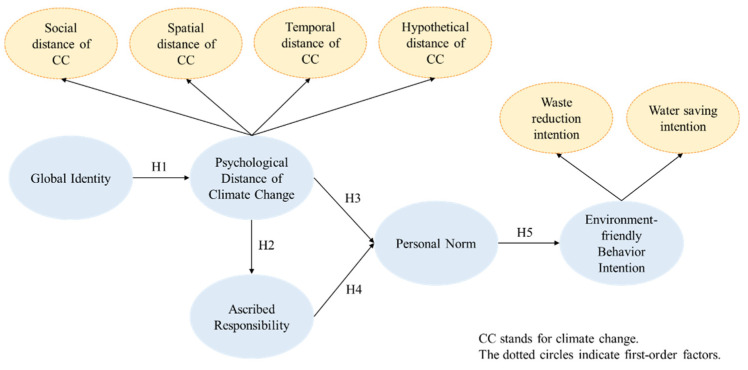
The suggested conceptual model.

Figure 1 graphically illustrates the study constructs and hypotheses suggested above.

## 3. Method

### 3.1. Measures and Questionnaire

Validated measures were adopted from previous studies to assess the research variables. Multiple measurement items were utilized to evaluate the constructs in the conceptual model. Specifically, three items were adopted to measure global identity (i.e., “I care about knowing global events (*β* = 0.802)”; “I identify myself as a global citizen (*β* = 0.852)”; and “I believe people should be made more aware of how connected we are to the rest of the world (*β* = 0.795)”) [15]. To evaluate psychological distance of climate change, three items for each dimension were used [27]. “Climate change mostly affects people I do not know (*β* = 0.858)”; “serious consequences of climate change primarily impact other people (*β* = 0.892)”; and “climate change is a significant problem mainly for others (*β* = 0.900)” were used to measure social distance. “Climate change mostly affects other parts of the world (*β* = 0.857)”; “climate change is a significant problem mainly in distant locations (*β* = 0.866)”; and “impacts of climate change are primarily experienced in developing countries (*β* = 0.761)” were used to evaluate spatial distance. “Climate change effects will mostly occur in the future (*β* = 0.976)”; “serious consequences of climate change will be felt primarily in the future (*β* = 0.818)”; and “climate change will be more of a significant problem in the future compared with now (*β* = 0.554)” were employed to measure temporal distance. “I am uncertain whether the climate is changing (*β* = 0.803)”; “I am uncertain over the causes of climate change (*β* = 0.941)”; and “I am uncertain what the effects of climate change are (*β* = 0.968)” were used to assess hypothetical distance. Ascribed responsibility was measured with three items (i.e., “I feel jointly responsibility for eco-friendly activities (e.g., waste reduction and water saving) while staying at a hotel” (*β* = 0.838)”; “I feel jointly responsibility for the negative consequences of not practicing eco-friendly activities (e.g., waste reduction and water saving) while staying at a hotel (*β* = 0.920)”; and “I feel joint responsibility for the environmental pollution and ecological damage problems caused by not practicing eco-friendly activities (e.g., waste reduction and water saving) while staying at a hotel (*β* = 0.941)”) [28]. Three items were utilized to assess personal norm (i.e., “I feel an obligation to practice eco-friendly activities (e.g., waste reduction and water saving) while staying at a hotel (*β* = 0.839)”; “regardless of what other people do, because of my own values/principles I feel that I should behave in an environmentally friendly way while staying at a hotel (*β* = 0.892)”; and “I feel that it is important to engage in eco-friendly activities while staying at a hotel, reducing the harm to the environment (*β* = 0.924)”) [7]. Lastly, in order to assess environment-friendly behavior intention, two items for each dimension were used [7]. “The next time I stay at a hotel, I am willing to engage in recycling activities (e.g., sorting my waste into the right bins) (*β* = 0.693)”and “the next time I stay at a hotel, I will expend effort on reducing waste (e.g., decreasing food waste, minimizing the use of disposable products, and decreasing the consumption of plastic bottles/canned products/papers) (*β* = 0.863)” were used to measure waste reduction intention. “The next time I stay at a hotel, I am willing to engage in towel/linen reuse activities (*β* = 0.478)” and “the next time I stay at a hotel, I will expend effort on saving water (e.g., turning off the tap water while brushing my teeth and turning off the shower water while I am lathering soap) (*β* = 0.820)” were used to evaluate water saving intention. All the items were measured with a seven-point Likert scale from “strongly disagree” (1) to “strongly agree” (7). Including the measurement items mentioned above, the questionnaire was composed of a brief research introduction and queries for respondents’ demographic characteristics. A pre-test for the questionnaire was conducted with five graduate students from a hospitality program and three hotel industry professionals. Based on their feedback, a slight modification was made. Lastly, the questionnaire was finalized through the reviews of academic experts.

### 3.2. Data Collection and Respondent Profiles

To collect the data, an online panel survey was performed through an internet-based survey firm’s system. General hotel guests in South Korea were randomly selected from the firm’s database. An e-mail invitation for the survey with a brief research introduction was sent to them. Only those who have stayed at hotels within the last nine months were invited to answer the questionnaire by clicking the survey link. The survey was collected from 13 April 2021 to 17 April 2021. A total of 1036 people initially participated in the survey. Out of them, 393 respondents completed the survey. After eliminating inconsistent responses, a total of 387 samples remained for analysis. The respondents stayed at hotels in a variety of locations and categories in South Korea.

Among the 387 samples, 48.6% were male and 51.4% were female guests. On average, the respondents were 43.84 years old, ranging from 20 to 69 years old. A total of 21.7% were in their 50s, 20.7% were in their 40s, 19.9% were in their 30s, 19.4% were in their 20s, and 18.3% were in their 60s. In terms of education level, 63.3% held a bachelor’s degree, 15.0% held graduate school degrees or higher, 12.9% held a high school diploma, and 8.8% held an associate degree. Regarding monthly income, 36.7% reported an income lower than USD 2500, followed by between USD 2501 and 4500 (32.6%), USD 4501 and 6500 (16.5%), USD 6501 and 8500 (8.5%), and over USD 8500 (5.9%). When asked for their frequency of hotel stay, the respondents reported 2.2 times per year on average.

## 4. Results

### 4.1. Measurement Model Evaluation

To generate the measurement model and evaluate the quality of the data, a confirmatory factor analysis (CFA) was performed. As shown in Table 1, the model adequately fit the data (χ^2^ = 592.730 (*df* = 255, *p* < 0.001, χ^2^/*df* = 2.324); RMSEA = 0.059, CFI = 0.956, IFI = 0.956, and TLI = 0.948). All the measurement items were significantly loaded on their associated constructs at *p* < 0.001. The composite reliability of the measures ranged from 0.856 to 0.982, showing higher values than the recommended cutoff of 0.700 [28]. The values of average variance extracted (AVE) ranged from 0.609 to 0.966, exceeding the cutoff point of 0.500 [28]. Thus, the convergent validity of the measurement items was established. Lastly, the AVE value of each construct exceeded its squared correlations with the other constructs, indicating the adequate discriminant validity of the constructs [28].

### 4.2. Structural Model Analysis and Hypotheses Testing

**Figure 2:**
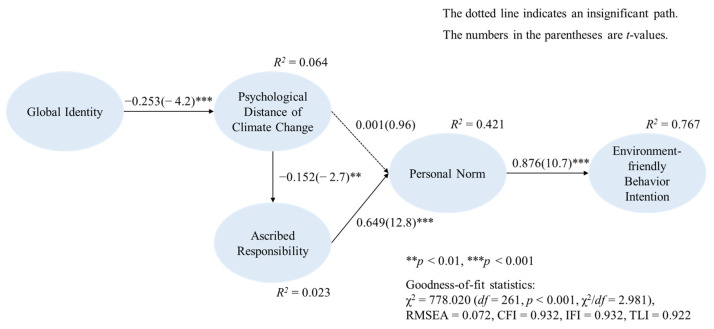
The structural model results.

To assess the hypothesized research model, structural equation modeling (SEM) was conducted. As shown in Table 2, the result revealed adequate goodness-of-fit statistics of the structural model to the data (*χ*^2^ = 778.020 (*df* = 261, *p* < 0.001, *χ*^2^/*df* = 2.981); RMSEA = 0.072, CFI = 0.932, IFI = 0.932, and TLI = 0.922). The model showed satisfactory variance explanation power for environment-friendly behavior intention (i.e., waste reduction intention and water saving intention) (*R*^2^ = 76.7%). The detailed results are shown in Table 2 and Figure 2.

Regarding the hypothesized relationships, as shown in Table 2 and Figure 2, the SEM results revealed that global identity significantly and negatively affected the psychological distance of climate change (*β* = −0.253, *p* < 0.001), supporting H1. In turn, the psychological distance of climate change showed a significant, negative effect on ascribed responsibility (*β* = −0.152, *p* < 0.01), supporting H2. On the other hand, the psychological distance of climate change did not significantly affect personal norm (H3) (*β* = 0.001, *p* > 0.05). However, a post hoc mediation test showed that the insignificant impact was due to the full mediation effect by ascribed responsibility. Specifically, when the path from ascribed responsibility to personal norm was constrained to zero, the impact of the psychological distance of climate change on personal norm became significant (*β* = −0.139, *t* = −2.37 (*p* < 0.05)). The χ^2^ difference between the original model and the constrained model (Δ*χ*^2^(1) = 205.74) was significant at *p* < 0.001, indicating that the mediation effect was highly significant. Going back to the original model, ascribed responsibility showed a significant, positive effect on personal norm (*β* = 0.649, *p* < 0.001) and, in turn, personal norm showed a significant, positive effect on environment-friendly behavior intention (*β* = 0.876, *p* < 0.001), supporting H4 and H5.

As a next step, the indirect effects of the constructs were assessed. As in Table 3, global identity showed a significant positive indirect effect on ascribed responsibility (*β* = 0.038, *p* < 0.05). The psychological distance of climate change exhibited a significant, negative indirect effect on personal norm (*β* = −0.099, *p* < 0.05). In turn, ascribed responsibility showed a significant positive effect on environment-friendly behavior intention (*β* = 0.568, *p* < 0.05). Lastly, in terms of the total effect on environment-friendly behavior intention, our findings indicated that personal norm exerted the greatest impact (*β* = 0.876, *p* < 0.05), followed by ascribed responsibility (*β* = 0.568, *p* < 0.01).

## 5. Discussion

### Discussions and Implications

As mentioned in the literature review, customers’ positive global identity has been suggested to reduce the psychological distance of climate change perception [5,12,17,19]. The findings of the current study empirically showed that global identity significantly reduced the psychological distance of climate change. It means the more that people feel part of the human group and the earth, the more that they psychologically closely recognize climate change. Thus, promoting that we (all humans) are globally connected, or stimulating hotel guests’ global identity, is helpful in inducing them to closely recognize climate change, thus making them ready to act. For example, hotels may display a campaign advertisement showing the beautiful scenery of the earth and saying that we are a part of it or belong to it. It would be more emotionally appealing to guests than directly warning about climate change because it touches their identity, which indicates where they are from [15]. Depending on hotels’ locations and target markets, various creative campaign ideas can be generated based on this finding. As such, this finding can be useful for hotels to make their guests feel that climate change is their own problem at hand.

The one of objectives of this research was to identify the relationships between the psychological distance of climate change, ascribed responsibility, and personal norm. Han et al. [7] noted the importance of tourists’ environmental responsibility. Chen [11] used construal level theory to analyze the psychological distance of climate change, tourists’ environmental concerns, and customers’ pro-environmental behaviors. This research disclosed that the psychological distance of climate change significantly reduced ascribed responsibility for not acting environment-friendly. It means that the more hotel guests perceive climate change will occur far in the future, away from them, to those who they do not know, and/or with a very low probability, the less they will perceive ascribed responsibility for not acting environment-friendly. Conversely, it implies that if hotels can lead their guests to perceive climate change as psychologically close, this perception can raise guests’ ascribed responsibility for not acting environment-friendly.

The norm activation model (NAM) theory is widely used in tourism research [10,21]. Prior studies have noted the importance of environment-friendly behavior [2,6,10]. This research set out to assess the extended norm activation model by global climate change background. Construal level theory explains that a change in one dimension of psychological distance accompanies changes in the other dimensions in the same direction [29]. It means that they are positively and closely correlated with each other, and thus, by reducing a distance in one dimension, distances can be reduced in other dimensions at the same time. Thus, if hotels could give guests a hint that climate change is temporally, spatially, socially, or hypothetically close to them, the guests would perceive climate change as psychologically close. For example, hotels may display campaign pictures of beautiful species of animals and breathtaking sceneries, saying that we are losing them forever, little by little every day, due to climate change (cf., [30]). Then, it would reduce guests’ temporal psychological distance of climate change, thus closing their psychological distances of climate change in other dimensions as well. Moreover, if an event is felt psychologically close, this feeling stimulates cognitive and emotional involvement with the event [31]. Therefore, hotels need to positively advertise to customers the knowledge of climate change and the importance of protecting the environment. Once climate change is felt psychologically close, it would induce guests to pay more cognitive and emotional attention to the harmful outcomes of climate change, and thus lead them to actively take actions to mitigate climate change.

## 6. Conclusions

Tested in the hotel industry context, this study revealed that guests’ ascribed responsibility for not acting environment-friendly positively influenced their personal norm for environment-friendly behavior. In turn, guests’ personal norm for environment-friendly behavior positively affected their environment-friendly behavior intention (i.e., waste reduction intention, water saving intention). These results are consistent with the findings of previous research using the NAM on other environment-friendly behaviors, such as sustainable transport behavior (e.g., [32]), energy conservation behavior (e.g., [33]), and environmental complaint behavior (e.g., [34]). Having been widely tested in various research contexts, the NAM is now accepted as a robust, rather-basic and essential model in explaining various pro-environmental behaviors [35,36]. Moreover, according to the literature research, the psychological distance of climate change is positively related to individual people’s environmental awareness [11,20]. The findings of this study suggest that perhaps the psychological distance of climate change would be a better predictor of ascribed responsibility, rather than simple awareness of consequences in pro-environmental behavior research contexts, since it reveals more specifically when, where, to whom, and in how much certainty the negative consequences are expected. By measuring those details, researchers can reveal more specific psychological processes of consumers’/citizens’ attitudinal and behavioral responses to climate change, and thus can draw more precise implications from their research findings.

### Limitations and Future Research

The samples in this study are restricted to Korean travelers who traveled during the COVID-19 pandemic period. Therefore, responses to the same questions can be different when asked to non-Koreans during post-COVID-19 period. Depending on cultural backgrounds, some people may have more holistic views of the world than others, leading them to having stronger global identity and thus feeling other people’s problems closer to themselves. The COVID-19 pandemic may have led people to feel that all humans are closely connected. In fact, we literally have affected one another regardless of any boundaries during the pandemic period. Thus, perhaps, during the post-COVID-19 period, people may develop stronger global identity than ever before and view the earth as one system where we have to rely on one another to survive. Therefore, the generalizability of the findings of this study may be further tested in other cultures during the post-COVID-19 period.

As mentioned earlier, future researchers may adopt the psychological distance of climate change instead of awareness of consequences when using or extending/modifying the NAM in explaining other types of pro-environmental behaviors to generate more specific findings. It appears that some people do not realize the apparent harmful outcomes of climate change although they are occurring in front of their eyes [37]. Therefore, it is a psychological perception that matters when we have to deal with climate change.

## Figures and Tables

**Table 1:** Results of the confirmatory factor analysis and correlations (*n* = 387).

Construct	(a)	(b)	(c)	(d)	(e)	CR	AVE
(a) Global identity	−	0.061 ^b^	0.274	0.419	0.375	0.857	0.667
(b) Psychological distance of climate change	−0.246 ^a^	−	0.019	0.005	0.022	0.856	0.609
(c) Ascribed responsibility	0.523	−0.138	−	0.480	0.408	0.928	0.811
(d) Personal norm	0.647	−0.072	0.693	−	0.748	0.916	0.784
(e) Environment-friendly behavior intention	0.612	−0.148	0.639	0.865	−	0.982	0.966
Mean	5.35	3.50	5.19	5.03	5.28		
*SD*	0.99	1.25	1.15	1.23	1.06		
Goodness-of-fit statistics: χ^2^ = 592.730 (*df* = 255, *p* < 0.001, χ^2^/*df* = 2.324), RMSEA = 0.059, CFI = 0.956, IFI = 0.956, TLI = 0.948							

Note: CR = composite reliability; AVE = average variance extracted; SD = standard deviation; RMSEA = root mean square error of approximation; CFI = comparative fit index; IFI = incremental fit index; TLI = Tucker-Lewis index; ^a^ Correlation; ^b^ Squared correlation.

**Table 2:** Results of the structural equation modeling (*n* = 387).

	Independent Construct		Dependent Construct	Coefficient	*t*-Value
H1	Global identity	→	Psychological distance of climate change	−0.253	−4.237 ***
H2	Psychological distance of climate change	→	Ascribed responsibility	−0.152	−2.716 **
H3	Psychological distance of climate change	→	Personal norm	0.001	0.975
H4	Ascribed responsibility	→	Personal norm	0.649	12.837 ***
H5	Personal norm	→	Environment-friendly behavior intention	0.876	10.733 ***
Total variance explained (*R*^2^): *R*^2^ for psychological distance of climate change = 0.064 *R*^2^ for ascribed responsibility = 0.023 *R*^2^ for personal norm = 0.421 *R*^2^ for environment-friendly behavior intention = 0.767				Goodness-of-fit statistics: χ^2^ = 778.020 (*df* = 261, *p* < 0.001, χ^2^/*df* = 2.981), RMSEA = 0.072, CFI = 0.932, IFI = 0.932, TLI = 0.922	

Note: RMSEA = root mean square error of approximation; CFI = comparative fit index; IFI = incremental fit index; TLI = Tucker-Lewis index; ** *p* < 0.01, *** *p* < 0.001.

**Table 3:** Results of the indirect and total effect assessment.

Indirect Effect of			
on	Global Identity	Psychological Distance of Climate Change	Ascribed Responsibility
Ascribed responsibility	0.038 *	–	–
Personal norm	0.025	−0.099 *	–
Environment-friendly behavior intention	0.022	−0.085	0.568 *
Total effect on environment-friendly behavior intention: *β* global identity = 0.022 *β* psychological distance of climate change = 0.085 *β* ascribed responsibility = 0.568 * β personal norm = 0.876 *			

Note: * *p* < 0.05.

## Data Availability

The dataset used in this research are available upon request from the corresponding author. The data are not publicly available due to restrictions, i.e., privacy or ethical.
